# Dataset of photovoltaic panel performance under different fault conditions cracks, discoloration, and shading effects

**DOI:** 10.1016/j.dib.2025.111392

**Published:** 2025-02-14

**Authors:** Bonie Johana Restrepo-Cuestas, Cristian Guarnizo-Lemus, John Alejandro Montoya-Marín, Jhon Montano

**Affiliations:** Department of Electronics and Telecommunications, Institución Universitaria ITM, Medellín 050028, Colombia

**Keywords:** Photovoltaic panels, Fault conditions, Shading effects, Thermographic images, Panel degradation, Energy production

## Abstract

This dataset presents the performance characteristics of photovoltaic (PV) panels under various fault conditions, including discoloration, cracks, and partial shading. The panels, SP090P Solar Plus Energy and HYBRYTEC-M5-30/12, were subjected to testing under three distinct scenarios: dirty surfaces, clean surfaces, and partial shading. Electrical parameters such as short-circuit current, open-circuit voltage, and output power, were measured, along with thermographic images to assess thermal performance under the specified conditions. Data was acquired using an electronic load system, oscilloscope, and thermographic camera, with testing conducted in outdoor environmental conditions. The results highlight the significant impact of faults and shading on the performance of PV panels, with notable reductions in power production. This dataset provides valuable insights into the real-world performance of PV systems and can serve as a reference for researchers focused on fault detection, optimization of maintenance strategies, and enhancing the overall efficiency of solar energy systems.

Specifications Table

**Table:** 

Subject	Renewable Energy, Sustainability and the Environment.
Specific subject area	Outdoor testing of solar PV panels under fault conditions: electrical and thermal data.
Type of data	**Raw data:** Temperature, irradiance, panel current, and panel voltage (.mat format); Thermal images (.csv, .IS2, and .png formats), and Visible Light (VL) images (.png format). **Processed data:** Temperature, irradiance, panel current, panel voltage, panel power, open circuit voltage V_oc_, short-circuit current I_sc_, and maximum power point P_mpp_ (.mat format); current-voltage curve and power-voltage curve (.SVG format); Thermal images (.csv and .pdf formats).
Data collection	These data were acquired experimentally in the Electronics and Renewable Energy laboratory in an outdoor environment. Date: May 17, 2024, from 10:00 am to 2:00 pm. Two different panel references were selected to acquire thermal and electrical information under various fault types (discoloration and cracks). **Testing Instruments:** i) Three photovoltaic panels with reference SP090P 90W (one without visual faults, one with cracks, and one with discoloration), ii) Three photovoltaic panels with reference HYBRYTEC M5-30/12 30W (one without visual faults, one with cracks, and one with discoloration). **Measuring Instruments:** i) BK precision 8514 electronic load and an oscilloscope with reference R\&S®RTE1204 to acquire the current-voltage curve of each panel, ii) Temperature and irradiance were acquired using a FLK-IRR1-SOL, and iii) Thermal images were acquired using a thermal camera Fluke Ti90. **Software**: The data processing was developed using Matlab.
Data source location	**Institution:** Institución Universitaria ITM. City: Medellín. Country: Colombia. Latitude and longitude: 6.244681, -75.552074.
Data accessibility	Repository name: Experimental data for Mismatching Faults of Photovoltaic Modules. Data identification number: 10.17632/xjs42j8dtf.2 Direct URL to data: https://data.mendeley.com/datasets/xjs42j8dtf/2
Related research article	None.

## 1 Value of the Data

• This dataset offers valuable insights into the performance of photovoltaic panels in real-world fault conditions, including discoloration, cracks, and shading. It also considers scenarios such as dirty and clean panel surfaces.
• The dataset includes thermographic images, visible light images, and detailed electrical data such as voltage, current, and power curves. This integration allows for a holistic analysis of PV panel performance.
• The selected panels have been in outdoor operation for more than 10 years, reflecting the performance of aged systems. This dataset can be used to develop and validate fault detection and classification algorithms. The combination of electrical and thermal data can also be used for building predictive models.

## 2 Background

In the realm of renewable energy, PV panel performance has gained significant attention throughout time [1,2]. Understanding how external elements like physical flaws, shade, and weather variables impact PV panel energy production is a major difficulty for solar energy systems. Problems like partial shade, discoloration, and cracks may significantly affect solar panel efficiency, impacting how well solar energy systems work as a whole [3,4].

This dataset was created to investigate the effects of typical defects, such as discoloration and cracks, on the electrical and thermal performance of PV panels in real-world scenarios [5]. The data was collected through experiments conducted under varying scenarios, including clean and dirty surfaces as well as partial shading, to replicate the environmental conditions photovoltaic panels encounter during regular use. Key electrical parameters, such as short-circuit current, open-circuit voltage, and power output, were recorded alongside thermographic images that allow for thermal performance analysis [6].

This information can assist the creation of diagnostic instruments and enhance methods for increasing the longevity and effectiveness of solar panels by examining how frequent defects impact performance.

## 3 Data Description

**Fig. 1:**
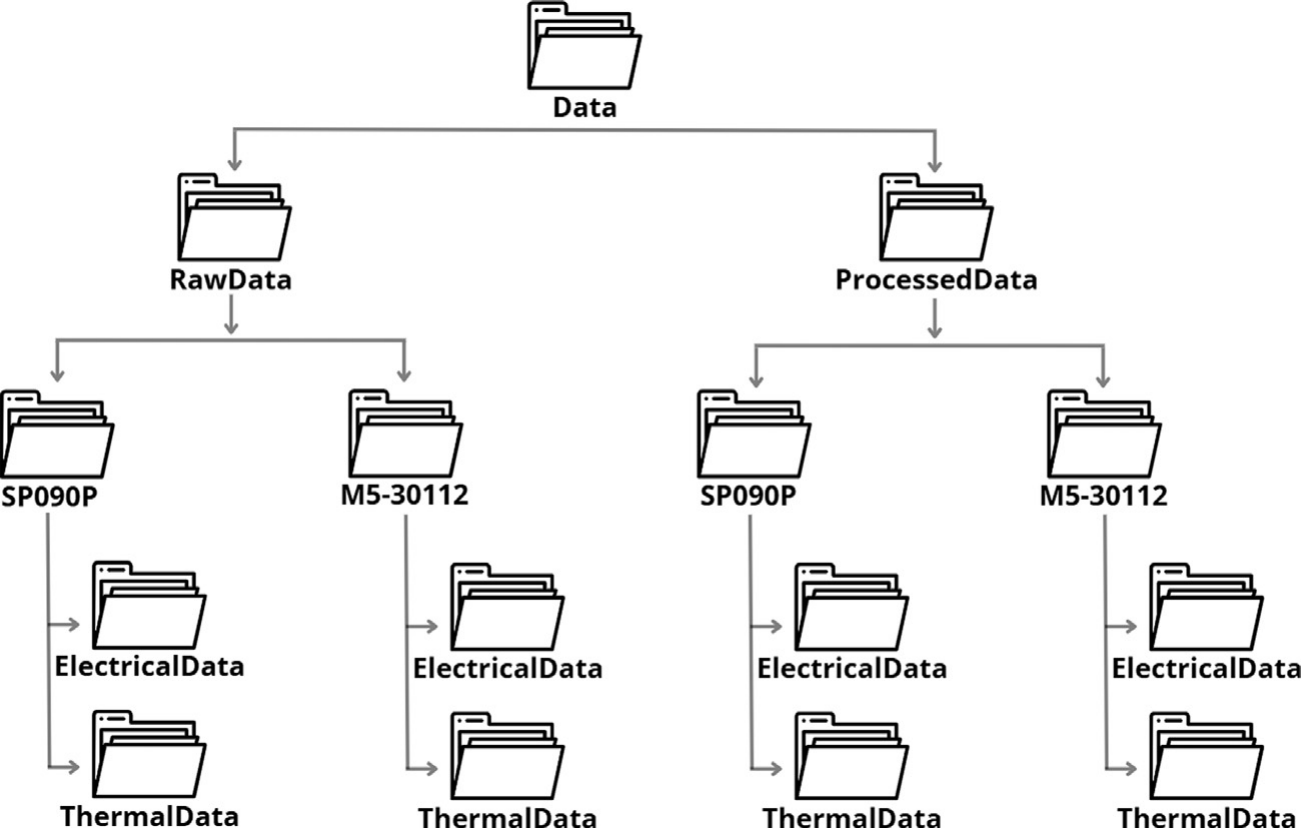
Distribution of folders in the database.

This article describes the database “Experimental Data for Mismatching Faults of Photovoltaic Modules”. The repository consists of electrical information (current-voltage curves) and thermal information (thermographic images) of two references of photovoltaic panels that have been used for more than 10 years in outdoor conditions. The information has been classified in two folders named “RawData'” and “ProcessedData'”. In each folder there is a subfolder for each panel reference, SP090P and M5-30112. In turn, each subfolder has two divisions: “ElectricalData” and “ThermalData” (see Fig. 1).

The “ElectricalData” folder contains the voltage [V] and current [A] vectors of the panel (Vpanel and Ipanel). In the case of raw data, this information is delivered by the oscilloscope, as well as the temperature data [°C] and irradiation [W/m^2^] measured at the time of the test (T and IRR). All data were consolidated in a file in .mat format.

In the case of the folder “ProcessedData”, the voltage [V] and current [A] data of the panel necessary to reproduce the I-V curve (Vpanel and Ipanel) are provided, as well as the power data [W] that allow reconstruction of the P-V curve (Ppanel), and some data of interest from the curves such as the open circuit voltage (Voc) [V], short circuit current (Isc) and maximum power point (Pmpp) [W]. All these data, as well as the temperature [°C] and irradiation [W/m^2^] measured at the time of the test (T and IRR), were saved in a file in .mat format. Each test's current-voltage and power-voltage figures are included (\_IV.SVG and \_PV.SVG).

The “ThermalData” folder, for raw data, delivers the information obtained through the thermal imaging camera in three different formats (.csv, .IS2, and .png). The .csv format provides an array of temperature data. The .IS2 format is proprietary to the camera manufacturer, and the .png format allows the test to be observed using the visible light spectrum.

In the case of processed data, the thermal information is presented in .csv and .pdf format after the figure area is adjusted to identify the point of highest temperature in the panel area.

For each panel reference, three units were selected: one that presents cracks, another that presents discoloration, and the third showing no failure upon visual inspection. The file names corresponding to each panel reference and each fault are consolidated in Table 1.

**Table 1:** Nomenclature for each panel reference and each fault.

Reference	Normal	Discoloration	Cracks
SP090P	Panel1	Panel2	Panel3
M5-30112	PanelA	PanelB	PanelC

**Fig. 2:**
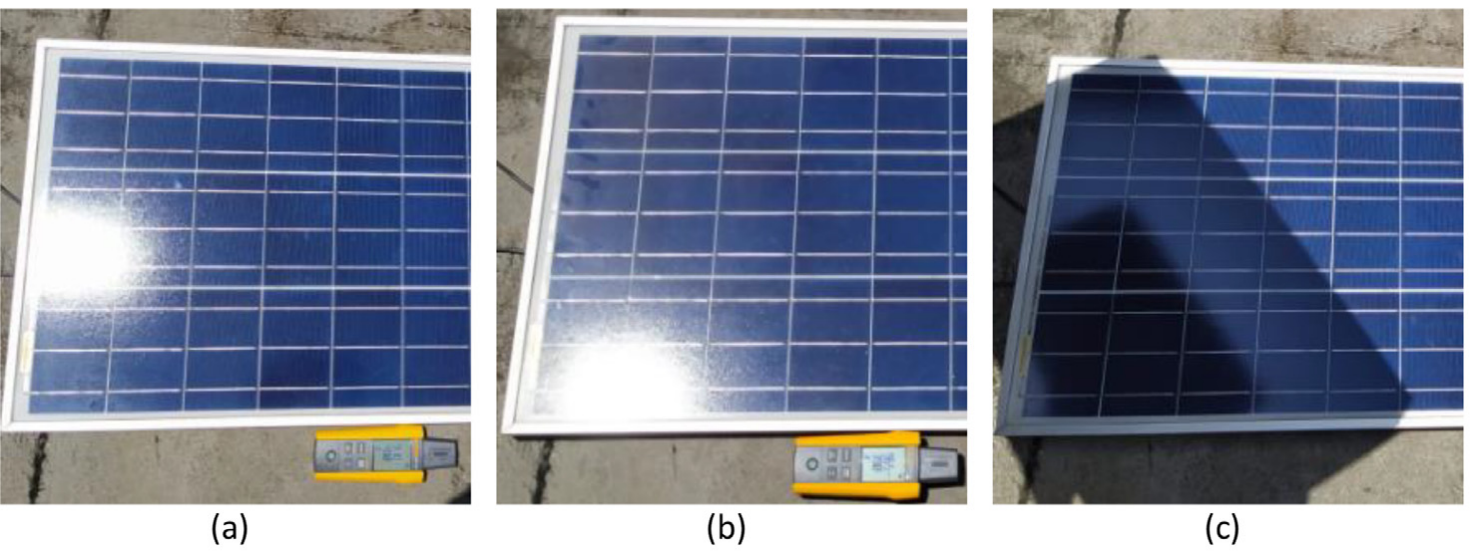
Panel 1 in different conditions: (a) Clean, (b) Dirty, and (c) under Partial Shading condition.

Afterward, three scenarios were proposed for the data acquisition in each of the six panels: (a) clean cell surface area, (b) dusty cell area, and (c) clean cell area and partial shading condition (see Fig. 2). In this case, the data for Panel1 and the clean surface are found in the file “Panel1\_Clean.mat”. In the case of the panel with the dusty surface in “Panel1\_Dirt.mat”, and finally for the test corresponding to partial shading in “Panel1\_Shadow.mat”.

## 4 Experimental Design, Materials and Methods

### 4.1 Materials

As already mentioned, two solar panel technologies were used as test panels. Three panels were selected and named from each technology as shown in Table 1. The datasheet information of both references is consolidated in Table 2.

**Table 2:** Datasheet information for selected photovoltaic panels references.

	SP090P Solar Plus Energy	HYBRYTEC-M5-30/12 ERDM SOLAR
Maximum power point (***P_mpp_***)	90 W	30 W
Current at ***P_mpp_*** (***I_mpp_***)	5 A	1.74 A
Voltage at ***P_mpp_*** (***V_mpp_***)	18 V	17.2 V
Shortcircuit current (***I_sc_***)	5.41 A	1.93 A
Open circuit voltage (***V_oc_***)	22.5 V	21.6 V
Number of cells per panel	36	36

**Fig. 3:**
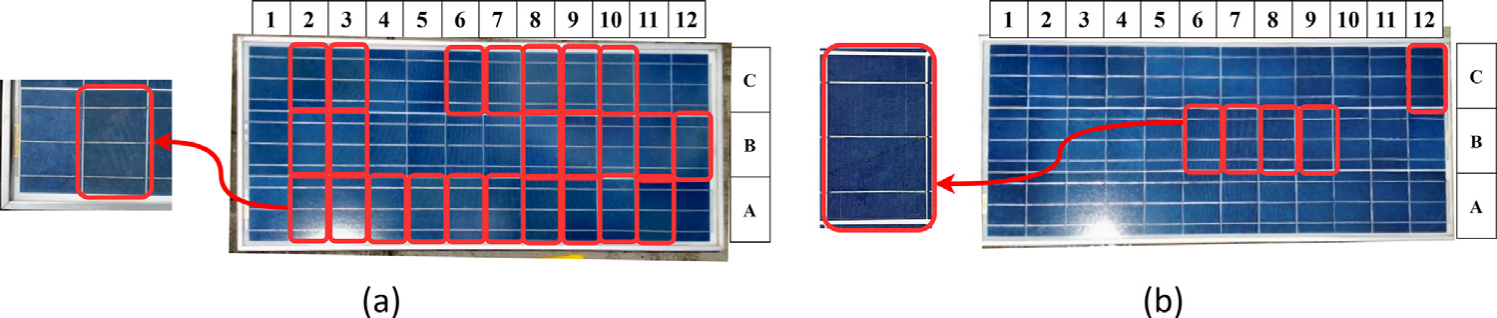
Faulty cells in: (a) Panel 2 and (b) Panel 3.

Fig. 3(a) presents a panel diagram where the cells identified as discolored are marked in red rectangles for Panel 2. Fig. 3(b) represents the cells affected by cracks in Panel 3.

**Fig. 4:**
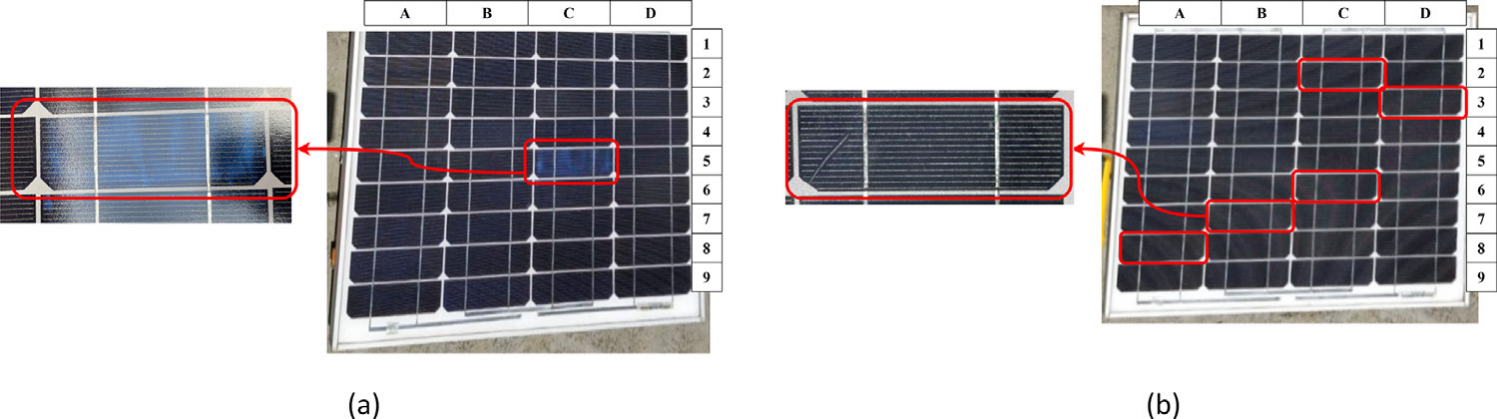
Faulty cells in: (a) Panel B and (b) Panel C.

For the SP090P reference, Fig. 4 shows the cells that present a failure, in the case of Panel B of the discoloration type and Panel C of the cracking type.

### 4.2 Acquisition of electrical and thermal variables

**Fig. 5:**
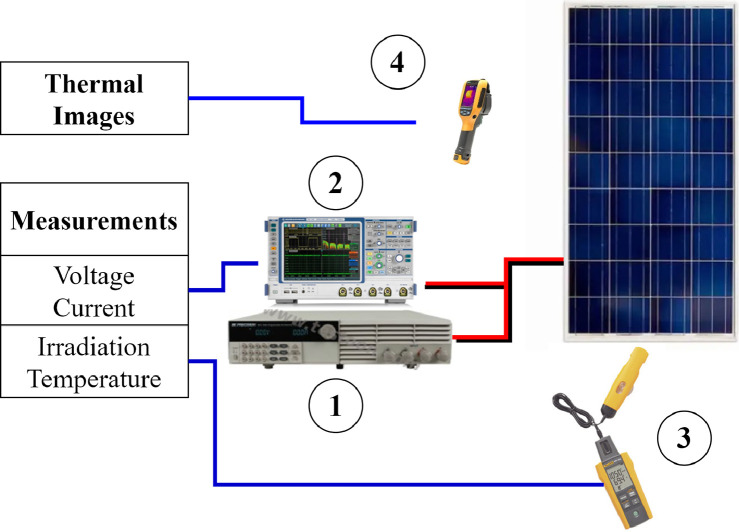
Test scheme for acquisition of electrical and environmental variables.

The tests were carried out using the test scheme to acquire electrical and thermal information for each selected panel (see Fig. 5). To acquire the current-voltage curves (I-V curves), it was necessary:

• To use a BK precision 8514 electronic load, as a tracer curve ①. In this case, a voltage sweep in constant voltage mode was programmed through serial communication.
• The current and voltage panel signals were acquired through an oscilloscope with reference R\&S^Ⓡ^RTE1204 ②.
• The irradiance and temperature were read using the FLK-IRR1-SOL solar irradiance measuring kit ③.
• Panel surface thermographic images were obtained using a Ti90 reference thermographic camera ④.

Regarding the SP090P technology panels, Panel 1 (no faults), Panel 2 (discoloration), and Panel 3 (cracks), the temperature and irradiation measurements are shown in Table 3. For the partial shading scenario, irradiance and temperature measurements were taken in both the shaded and unshaded regions.

**Table 3:** Environmental information at the time of acquisition of the I-V curves for each panel with SP090P technology.

Condition	Panel 1		Panel 2		Panel 3	
	Irr [W/m^2^]	T [°C]	Irr [W/m^2^]	T [°C]	Irr [W/m^2^]	T [°C]
Dirt	970	39.8	970	39.9	935	39
Clean	920	40.3	950	39.6	933	39.3
SP (non-shaded area)	942	43.2	946	41.4	940	41.9
SP (shaded area)	82	43.2	100	40.3	80	40.8

In the same way, the tests for the panels with reference HYBRYTEC-M5-30/12 were carried out is Table 4. Table 3 consolidates the test data by panel and by test scenario. The tests were performed consecutively to obtain similar irradiation and temperature values for each scenario.

**Table 4:** Environmental information at the time of acquisition of the I-V curves for each panel with HYBRYTEC-M5-30/12 technology.

Condition	Panel A		Panel B		Panel C	
	Irr [W/m^2^]	T [°C]	Irr [W/m^2^]	T [°C]	Irr [W/m^2^]	T [°C]
Dirt	900	39.4	950	40.3	990	37.4
Clean	910	42.6	948	40.1	900	34.2
SP (non-shaded area)	934	42.7	970	39.9	900	37.9
SP (shaded area)	134	41.4	130	39.5	80	37.9

### 4.3 Data processing

**Fig. 6:**
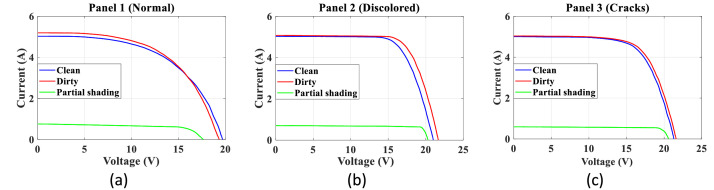
I-V Curves for SP090P panels.

The oscilloscope generated a .wfm file containing one million points of current and voltage as a function of time. The total number of samples that allowed visualization of at least three I-V curves were acquired to guarantee no considerable changes in irradiation during the acquisition. These data were converted into column vectors in MATLAB, where the corresponding I-V curves were plotted. Then, a range of data was selected to extract only the information of a single curve. Subsequently, using the trapezoidal integral could reduce the dataset to 300 points, preserving the curve's shape. The obtained curves were interpolated to determine the ***V_oc_*** and ***I_sc_*** values. Finally, the power panel was calculated as ***P_panel_=V_panel_∙I_panel_***, and the values related to the maximum power point were calculated (***P_mpp_, V_mpp_***, and ***I_mpp_***). Fig. 6 presents the I-V curves for SP090P panels.

Environmental information at the time of acquisition of the I-V curves for each panel with HYBRYTEC-M5-30/12 technology.

**Fig. 7:**
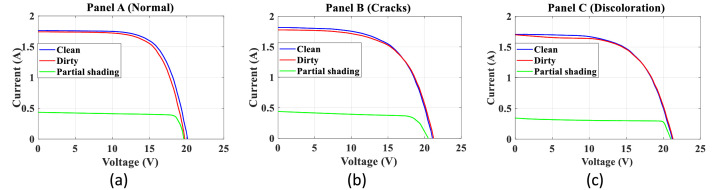
I-V curves for HYBRYTEC-M5-30/12 panels.

Fig. 7 presents the I-V curves for HYBRYTEC-M5-30/12 panels. To find this figure corresponding to Panel A in the database, follow the path “ProcessedData/M5\_30112/ElectricalData”.

**Fig. 8:**
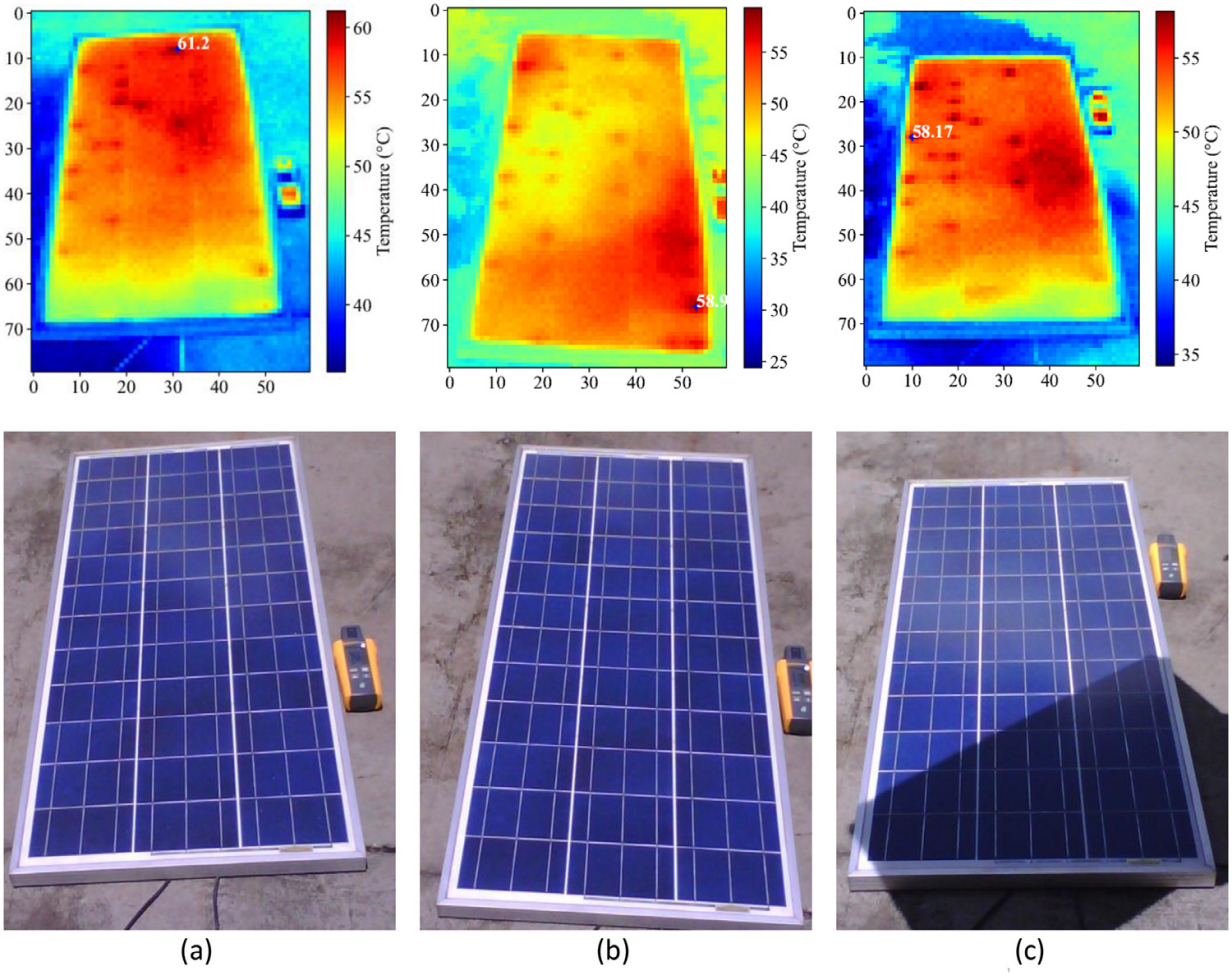
Thermography for Panel 1 in different state conditions.

Additionally, thermography pictures for each state and each panel were acquired. The results for Panel 1 are shown in Fig. 8. It is observed that when the panel was dirty, the maximum temperature was 61.20°C. Subsequently, with the panel clean, a uniform heat distribution is observed with a peak of 58.97°C. Finally, with partial shading, the highest temperatures occur at the top, where it is not shaded. In the processed data folder, the raw csv files were processed by filtering the panel area for correct indication of the highest temperature point.

## Limitations

Not applicable.

## Ethics Statement

The authors have read and follow the ethical requirements for publication in Data in Brief and confirming that the current work does not involve human subjects, animal experiments, or any data collected from social media platforms.

## Credit Author Statement

**Bonie Johana Restrepo-Cuestas:** Conceptualization, Methodology, Writing - Original draft, Data acquisition, Validation, Formal analysis, and Investigation; **Cristian Guarnizo-Lemus:** Conceptualization, Methodology, Writing - Original draft, Software, Formal analysis, Data acquisition, and Investigation; **John Alejandro Montoya-Marín**: Writing - Original draft, Investigation, and Data Curation, Review & Editing; **Jhon Montano:** Conceptualization, Investigation, Writing - Original draft, Data curation, Investigation, Formal analysis, Review & Editing.

## Data Availability

Mendeley DataExperimental data for Mismatching Faults of Photovoltaic Modules (Original data).

## References

[1] Praveen J., Vijaya Ramaraju V. (Jan. 2017). Materials for optimizing efficiencies of solar photovoltaic panels. Mater. Today Proc..

[2] Shaik F., Lingala S.S., Veeraboina P. (Apr. 2023). Effect of various parameters on the performance of solar PV power plant: a review and the experimental study. Sustainable Energy Res..

[3] Aslam A., Ahmed N., Qureshi S.A., Assadi M., Ahmed N. (Oct. 2022). Advances in solar PV systems; a comprehensive review of PV performance, influencing factors, and mitigation techniques. Energies 2022.

[4] Oni A.M., Mohsin A.S.M., Rahman M.M., Hossain Bhuian M.B. (Jun. 2024). A comprehensive evaluation of solar cell technologies, associated loss mechanisms, and efficiency enhancement strategies for photovoltaic cells. Energy Reports.

[5] Hayat M.B., Ali D., Monyake K.C., Alagha L., Ahmed N. (Mar. 2019). Solar energy—a look into power generation, challenges, and a solar-powered future. Int. J. Energy Res..

[6] Kim J.Y., Lee J.W., Jung H.S., Shin H., Park N.G. (Aug. 2020). High-efficiency perovskite solar cells. Chem. Rev..

